# Protocol: Interventions aimed at preventing out‐of‐home placement of children: A systematic review

**DOI:** 10.1002/cl2.1395

**Published:** 2024-04-10

**Authors:** Nina Thorup Dalgaard, Anja Bondebjerg, Elizabeth Bengtsen, Jens Dietrichson, Anders Bach‐Mortensen

**Affiliations:** ^1^ VIVE – The Danish Center for Social Science Research Copenhagen Denmark; ^2^ Social Policy and Intervention University of Oxford Oxford UK; ^3^ Roskilde University Roskilde Denmark

## Abstract

This is the protocol for a Campbell systematic review. The objectives are as follows. The aim of the present review is to synthesize evidence on the effectiveness of interventions for at‐risk families aimed at preventing the out‐of‐home placement of children or increasing the likelihood that children are reunited with their birth families following temporary care arrangements. The review has two objectives: (1) To assess the effectiveness of interventions for at‐risk families with children aged between 0 and 17 years old on measures of out‐of‐home placement and on secondary outcomes. (2) To identify factors that modify intervention effectiveness (e.g., prior placements, parental risk factors such as substance abuse, mental health issues, age, minority status, child risk factors such as disabilities, age, and gender).

## 1 BACKGROUND

### 1.1 The problem, condition or issue

As of 2017, it was estimated that 2.7 million children were placed in out‐of‐home care (Petrowski et al., 2017). Children in families with parents that are vulnerable and/or experience significant adversity due to factors such as teenage parenthood, poverty, unemployment, alcohol or substance abuse, lack of parental role models and social support, or domestic violence, are at increased risk of being placed in out‐of‐home care (Esposito et al., 2013; Farmer et al., 2008; Franzén et al., 2008; Storhaug & Kojan, 2017).

Placing children in out‐of‐home care is a costly intervention, and longitudinal studies suggest that children placed in out‐of‐home care are at increased risk of maladaptive development and adverse outcomes in adulthood, when compared with children who grow up in their family of origin (Clausen et al., 1998; Olsen et al., 2011; Pandiani et al., 2001; Vinnerljung, Hjern, et al., 2006; Vinnerljung & Hjern, 2014; Vinnerljung & Ribe, 2001; Vinnerljung & Sallnäs, 2008). In a systematic review of 20 Scandinavian studies in which children placed in out of home care were compared with children from the general population, Kääriälä & Hiilamo (2017) included studies reporting on 9 types of adverse outcomes: educational challenges, self‐supporting problems, mental health problems, criminality, suicidal behavior, teenage parenthood, mortality, alcohol and substance use, and disability pension. Based on a narrative synthesis, the overall conclusion was that placement in out‐of‐home care in childhood, when compared with those who were never placed in out‐of‐home care, appears to be consistently associated with negative outcomes in young adulthood in each outcome category across the studies. The authors further stated that these results held true after adjusting for birth parents' various socio‐economic, demographic, and mental health‐related factors. However, placing a child in out of home care most likely reflects the child's pre‐existing risk factors, so it is possible that the placement in out of home care prevented even further deterioration (Kääriälä & Hiilamo, 2017).

Although placing a child in out‐of‐home care may be seen as the ultimate intervention to ensure that children receive adequate care, some studies suggest that a care placement alone does not always decrease the child's risk of later maladaptive development. Vinnerljung (1997) reviewed approximately 50 long‐term follow‐up studies of foster children in adult age, and found that outcomes tended to resemble those of at‐risk peers who had grown up in their birth families, almost regardless of the outcome measured. However, placing a child in out‐of‐home care is often the last resort for children in families with multiple difficulties, and in some instances no change in the child's exhibited symptom levels or behavioral problems following out‐of‐home placement may be seen as a positive outcome, as a prolonged stay in the birth home might have brought about an even worse prognosis. Several studies report little to no differences in outcomes between children placed in out‐of‐home care compared to children who were referred to but received no or other services by the same agency (Lindsey, 2019; Parton, 1987; Vinnerljung, Sundell, et al., 2006).

In a meta‐analysis of outcomes for children in out‐of‐home care (*k* = 11), it was found that the symptoms and behavior problems that the children faced at the time when they were placed in out‐of‐home care did not on average decrease over time (Goemans et al., 2015). In a subsequent review and meta‐analysis (*k* = 31), Goemans et al. (2016) compared children in foster care with children at risk who remained with their biological parents and with children from the general population. Findings from the meta‐analysis suggest that on measures of cognitive, adaptive, and behaviorial functioning, children at risk who remained within their biological families, and children placed in foster care, show similar levels of problems and symptoms while there are significant differences between these two groups of children and children from community samples, who display significantly fewer developmental problems.

These findings may be interpreted in different ways. At face value, the findings may suggest that out‐of‐home placement in itself is ineffective as children in out‐of‐home care display more negative symptoms than children in community samples, and children in care display the same level of problems as children who faced similar risks but remained with their birth families and were provided with supportive interventions within the home. However, most of the existing studies suffer from a potential confounding bias, as the decision to place a child in out‐of‐home care is not random, and thus children in foster care and children, who are allowed to remain with their biological families and receive in‐home services are perhaps not comparable. Children placed in out‐of‐home care have often experienced significant adversity such as abuse and neglect before the out‐of‐home placement, and the placement may thus have prevented further deterioration or increases in their symptoms.

As an example, Baldwin et al. (2019) compared three groups of children involved with child welfare services due to maltreatment. The first group consisted of children currently in foster care, the second group included children who had previously been in care but had been reunified with their birth families, and the last group represented children who had never been in care but had received in‐home services. The study included a large set of potential confounders. Adjusting for the confounders that were significantly associated with both placement in care and outcomes, the odds of having a mental health problem were not significantly different whereas the odds of having a reactive attachment disorder were significantly higher for children who were currently placed in care compared to children who were never in care. However, as the authors discuss, despite the detailed data on confounders, selection bias is difficult to rule out and the estimates might not reflect the causal effects of out‐of‐home care.

A set of studies from the United States and Canada using the quasi‐random assignment of caseworkers to child maltreatment investigations as a natural experiment comes perhaps closest to estimating the unbiased effects of out‐of‐home care (Lindquist, 2023). This empirical strategy, pioneered by Doyle (2007, 2008), utilizes that caseworkers have different tendencies to recommend out‐of‐home placement, which, combined with the quasi‐random assignment to investigations, enables the use of the caseworker tendency as an instrumental variable for out‐of‐home placement. The resulting local average treatment effect is the effect for children at the margin of being placed in care – that is, children for whom caseworkers might reasonably disagree on whether they should be placed or not, which is a policy relevant subgroup (Doyle & Aizer, 2018; Bald, Doyle, et al., 2022).

The effects of out‐of‐home placement are quite heterogeneous in this set of studies. For example, some studies found harmful effects on crime and delinquency (Doyle, 2008; Roberts, 2019), teen motherhood, unemployment, and earnings (Doyle, 2007), as well as educational outcomes (Roberts, 2019; Warburton et al., 2014). Other studies found beneficial effects of out‐of‐home placements on crime (Baron & Gross, 2022; Warburton et al., 2014), and educational outcomes (Bald, Chyn, et al., 2022; Gross & Baron, 2022; Roberts, 2019). There is also substantial heterogeneity between subgroups of children within and between these studies. For example, effects are typically considerably more beneficial on all types of outcomes for children placed at a younger age (Bald, Chyn, et al., 2022; Baron & Gross, 2022; Doyle, 2007, 2008; Gross & Baron, 2022; Roberts, 2019). Effects on delinquency/crime are more harmful for boys in Roberts (2019) but more beneficial for boys in Baron & Gross (2022), and the beneficial effects on educational outcomes are only large and statistically significant for young girls in Bald, Chyn, et al. (2022).

Plausible explanations for the heterogeneous effects include differences across contexts in the type of placement, the reason for the placement, and the length and stability of placement (Bald, Chyn, et al., 2022; Baron & Gross, 2022; Gross & Baron, 2022; Lindquist, 2023). Family home placements are thought to be better for children than placement in institutions (Baron & Gross, 2022; Gross & Baron, 2022), which may in some cases be criminogenic (Lindquist, 2023). Young children are typically in out‐of‐home care to protect them from parental abuse or neglect whereas adolescents are more often removed from their homes for reasons that (also) have to do with, for example, their own criminal activity or addiction (Lindquist, 2023; Roberts, 2019). Shorter and more stable placements seem to produce better outcomes and shorter placements indicate that families are reunited faster (Baron & Gross, 2022; Gross & Baron, 2022). The results in the studies by Baron and Gross suggest that family reunifications and the improvements made by birth parents to the home environment are important explanations of the beneficial effects of out‐of‐home placements in the context they study.

From a psychological perspective, the loss of or separation from caregivers is traumatic for the child and may lead to subsequent attachment difficulties and increased psychological vulnerability (Bruce et al., 2019). Similarly, having a child placed in out‐of‐home care is often a traumatic experience for the parents (Storhaug & Kojan, 2017). Removing a child from the family of origin is not just a costly intervention from an economic perspective; it also comes with a great risk of iatrogenic effects for the intervention recipients.

From a legal perspective, it may constitute a human rights violation of both the child and the birth parents when the authorities fail to provide support for vulnerable families. This is often the case when authorities place the child with a foster family and restrict parental access to the child leading to a situation in which the authorities authorize the foster families to adopt the child based on the lack of an emotional bond between the biological parents and the child. In recent years, the European Court of Human Rights has ruled in support of birth families where the authorities have placed children in foster or adoptive families. Strand Lobben and Others v. Norway 2019 (Application no. 37283/13) is considered a key case and the judgment is useful for understanding the Court's contemporary position on out‐of‐home placement and adoption without consent from biological parents. In Strand Lobben and Others v. Norway, the Court reiterates that there is a broad consensus, including in international law, in support of the idea that in all decisions concerning children, the child's best interests are of paramount importance. Furthermore, the Court emphasizes that in cases involving the care of children and contact restrictions, the child's interests must come before all other considerations. At the same time, the Court states that it should be noted that regard for family unity and for family reunification in the event of separation are inherent considerations in the right to respect for family life under Article 8 of the European Convention on Human Rights. Accordingly, in the case of imposition of public care restricting family life, a positive duty lies on the authorities to take measures to facilitate family reunification as soon as reasonably feasible.

Generally, the best interests of the child dictate that the child's ties with its family must be maintained, except in cases where the family has proven to be particularly unfit. It follows that family ties may only be severed in very exceptional circumstances, and that everything must be done to preserve personal relations and, if and when appropriate, to “rebuild” the family. Another guiding principle is that a care order should be regarded as a temporary measure, to be discontinued as soon as circumstances permit, and that any measures implementing temporary care should be consistent with the ultimate aim of reuniting the natural parents and the child (European Court of Human Rights, 2019).

Similarly, Article 3 of the United Nations Convention on the Rights of the Child states that in all decisions and actions that concern children, the best interests of the child shall be a primary consideration, and in Article 9 it is mandated that no child should be separated from their parents against their will – unless it is in the child's best interests. These articles have clear legal implications for the placement of children in out‐of‐home care, as authorities are legally obliged to prevent out‐of‐home placement and promote family reunification whenever this is considered to be in the best interest of the child.

In summary, some longitudinal studies of children in out‐of‐home care suggest that out‐of‐home placements may be associated with an increased risk of adverse outcomes later in life and in adulthood. Previous research also suggests that when compared to children living with their family of origin, children placed in out‐of‐home care show more developmental and mental health problems. The studies that come closest to estimating the causal effects of out‐of‐home care indicate heterogeneous effects across contexts. Thus, the lack of positive development for children in out‐of‐home care may be caused by the adversity that the children faced before being placed in care in some contexts, and a consequence of the placement in out‐of‐home care in other contexts.

This suggests that placement in out‐of‐home care prevents further deterioration in highly vulnerable children or that placement in out‐of‐home care is not a more effective intervention than in‐home efforts. However, placing a child in out‐of‐home care is a life altering event for the child and the family and should only happen when no other intervention efforts are possible. Besides being a costly intervention, placement in out‐of‐home care is also a psychologically traumatic event for both the child and the birth parents and may in some cases constitute a human rights violation. While placement in out‐of‐home care may in some instances be the most appropriate intervention, authories have an ethical and legal obligation to prevent parents and children from being unneccesarily separated. Therefore, it is of fundamental importance to gather and synthesize existing evidence on the effects of preventive interventions on measures of out‐of‐home placement of children in at‐risk families, which is where the present review will contribute.

### 1.2 The intervention

In this review, we aim to examine the effectiveness of all kinds of efforts and interventions made to prevent children from being placed in out‐of‐home care or to promote children being reunited with their birth parents following temporary care arrangements.

That is, we will include studies of the effects of any kind of support or service that authorities or others may offer at‐risk families to decrease the risk of children being taken into care and placed outside their home. Furthermore, we will include family reunification interventions aimed at supporting parents in regaining custody of children placed in temporary care arrangements. In the following, we will elaborate on our choice of including interventions aimed at both preventing out‐of‐home placement and promoting family reunification, as well as our inclusion of secondary outcomes at both the child, parent, and family level.

As noted in Section 1.1, a number of studies have found heterogenous effects of out‐of‐home placement, pointing to a risk of harmful effects of this type of placement on children in some contexts. Although out‐of‐home placement is necessary in cases of severe child maltreatment, the fact that out‐of‐home placement may be harmful for children suggests that there is also room for effective prevention. Therefore, one of the goals of the present review is to provide knowledge about what interventions effectively prevent out‐of‐home placements. Such knowledge is of particular importance in countries like the United States, where the Family First Prevention Services Act of 2019 recently made it possible for states to use federal funding on services designed to prevent out‐of‐home placement (Bald, Doyle, et al., 2022).

Further, plausible explanations of the heterogeneous effects of out‐of‐home placements suggest an important role for family reunification and relatively short placements (Baron & Gross, 2022; Gross & Baron, 2022). These results motivate us to include interventions that promote family reunification in this systematic review.

In relation to outcomes, some studies suggest that when there are beneficial effects of out‐of‐home placements, they typically appear years after the placement has ended (Baron & Gross, 2022; Gross & Baron, 2022). This pattern indicates that improvements to the home environment by birth parents are an important factor for the effects. Such improvements are in turn an indication that parental behavior is amenable to intervention. It follows that if effective preventive interventions can induce improvements to the home environment before any placement, child and parent suffering associated with out‐of‐home placements might be avoided. Therefore, our review will examine the effects of preventive interventions not only on measures related to the child, but also on parental and family outcomes.

Additionally, Baron and Gross (2022) found no effects on crime of community and targeted services *without* placement. That is, the threat of placement might be a key factor influencing parental behavior and in turn for improvingchild outcomes. However, as discussed by Baron and Gross, it may also be the case that parents of children in out‐of‐home care receive different services than parents with children still at home. In any case, these results provide further motivation for our investigation of a range of secondary outcomes. If an intervention effectively prevents out‐of‐home care but affects, for example, child well‐being negatively, then the intervention may be harmful on balance.

The interventions included in this review may contain a number of different components such as case management, parental or child individual psychotherapy, multisystemic therapy (MST), family therapy, housing assistance, 24‐h on‐call availability, weekend family homes, financial assistance, leisure activities, home consultants, in‐home services, family foster care or family institutional placement, support persons, and mentoring (Lee et al., 2014; Storhaug & Kojan, 2017). We expect that most interventions will include multiple components and that many interventions offer services at an ad hoc basis, meaning that all families who receive the intervention may not receive exactly the same treatment components as a part of the treatment approach is to meet the specific needs of each family. We will include all such studies, as the aim of the review is to offer a comprehensive overview of existing preventive interventions. The list of possible services mentioned in the protocol may not be exhaustive.

### 1.3 How the intervention might work

Child abuse and neglect are complex problems that can have serious and long‐lasting consequences for children's physical, emotional, and psychological wellbeing (Norman et al., 2012). While the causes of child maltreatment are multifaceted and varied, a number of risk factors have been identified as increasing the likelihood of child abuse and neglect. It is important to note, however, that while these risk factors can increase the likelihood of child maltreatment, they do not mean that parents are inherently abusive or neglectful. In fact, most parents want the best for their children and are doing the best they can with the resources and support available to them.

Some of the most common risk factors for child abuse and neglect include parental stress, substance abuse, mental health problems, poverty, social isolation, lack of support, and inadequate parenting skills (Chaffin et al., 2004). For example, parents who are struggling with substance abuse or mental health issues may have difficulty providing adequate care and supervision for their children, while parents who are socially isolated may lack the support and resources needed to cope with the demands of parenting.

Preventive interventions aimed at preventing out‐of‐home placement of children are designed to help parents acquire the necessary skills, knowledge, and resources to provide a safe and nurturing environment for their children. These interventions typically focus on improving parenting practices, strengthening family relationships (Hurlburt et al., 2013), and addressing underlying issues such as poor mental health, substance abuse, and domestic violence that may be impacting the family's ability to function (Neo et al., 2021). Interventions aimed at addressing parental risk factors and problem behaviors may also include parent education programs, home visitation programs, and family support services. These interventions work by supporting parents in multiple ways and are often multi‐component interventions in which caseworkers aim to tailor the interventions to the specific needs of the families.

One of the most well‐known interventions is Parent–Child Interaction Therapy (PCIT), which has been shown to reduce child behavior problems and prevent child maltreatment amongst families at risk for out‐of‐home placement (Eyberg et al., 2008). PCIT is a short‐term treatment that combines play therapy and behavioral therapy techniques to teach parents how to communicate effectively with their children, set limits and boundaries, and provide positive reinforcement for appropriate behavior.

Another example of an intervention is the Incredible Years program, which aims to improve parent‐child interactions, reduce child behavior problems, and prevent child maltreatment (Hurlburt et al., 2013). The Incredible Years program is a comprehensive parenting intervention that focuses on strengthening positive parenting practices, promoting children's social‐emotional competence, and reducing harsh and inconsistent parenting. The program consists of group‐based parent training sessions, child social skills training sessions, and teacher training sessions.

A number of factors may moderate the effects of interventions. We will include many intervention types, which will likely have different duration, intensity, or, more generally, different components. Although learning whether some intervention types have larger effects than others is very interesting, we do not know what interventions we will include. It is therefore difficult to describe comprehensively how they might work and to prespecify a confirmatory moderator analysis of intervention types. We will examine the effect sizes across intervention types or components in exploratory moderator analyses instead.

Other potential moderators relate to study design and risk factors. We will include both randomized controlled trials (RCTs) and quasi‐experimental studies (QESs). As these study designs have different strengths and weaknesses relating to the internal and external validity of the effect estimates, the study design may explain effect size heterogeneity.

Studies have repeatedly found intergenerational effects of family violence, mental health issues, teenage parenthood, and alcohol/substance abuse, and thus parents who as children were placed in out‐of‐home care due to these risk factors may be less likely to benefit from interventions than parents who grew up with their birth families without these risk factors (Brännström et al., 2022). Similarly, parents who have previously had older children placed in out‐of‐home care may also be less likely to benefit from interventions than parents who have not previously had a child placed in out‐of‐home care (Fuller, 2005). Furthermore, potential risk factors relating to the child may also moderate the effects of treatment. Thus, families in which the child has a disability may benefit less from interventions as caring for a disabled child may be more challenging for the parents (Rosenberg & Robinson, 2004; Lightfoot et al., 2011). The age, ethnicity, and gender of the child may also moderate treatment effects as these factors are associated with the risk of being placed in out‐of‐home care (Lu et al., 2004; Esposito et al., 2013).

Moderator and sub‐group analysis in previous reviews have found moderating effects of some of these factors (e.g., Al et al., 2012; Dijkstra et al., 2016; Macleod & Nelson, 2000; Maltais et al., 2019; see next section for more information about earlier reviews). However, except for study design, no moderator was significantly correlated with the effect sizes in more than one moderator analysis. Furthermore, several analyses used single‐factor subgroup analysis, which, as moderators are often associated, makes it difficult to learn the moderating effects of each moderator, conditional on the others. Thus, it is difficult to use earlier meta‐analyses to prioritize among moderators in a confirmatory moderator analysis. Although prognostic risk factors may not be strong moderators in some settings (Deeks et al., 2023), as earlier moderator analyses have found significant association between risk factors and effect sizes, we believe it is motivated to examine such factors in our case. Furthermore, a general theoretical reason to expect prognostic risk factors to moderate the effects in our setting is that the interventions will likely *reduce* the risk of out‐of‐home placement but not completely *remove* it. Therefore, even if interventions are on average relatively successful, there may be subgroups with very high risk for which the interventions have no effects.

### 1.4 Why it is important to do this review

Before the year 2000, numerous systematic reviews examined the effects of named family preservation interventions such as MST. These reviews have been criticized for lack of methodological rigor due to issues such as lack of transparency about inclusion criteria, lack of systematic strategies for locating relevant published and unpublished data, lack of clear standards used to evaluate evidence, and inappropriate methods used to synthesize results across studies (Littell, 2005, 2008). Furthermore, a systematic review of MST revealed a number of methodological errors within primary studies (Littell, 2005). Due to these issues, reviews published before the year 2000 will not be presented in the current protocol as the results may be biased and are in any case outdated.

A number of previous systematic reviews are relevant to the present review, but are focused on exploring the effects of named interventions, or they only include parents with a specific risk factor, such as mothers with substance abuse. We present these reviews next.

Dijkstra et al. (2016) explored the effectiveness of Family Group Conferencing (FGC) in youth care by conducting a systematic review and a meta‐analysis, which included 14 controlled studies (*N* = 88,495 participants). Child safety defined as reports of child maltreatment and out‐of‐home placement and involvement of youth care were included as outcome variables. Overall, results showed that FGC did not significantly reduce child maltreatment, out‐of‐home placements, and involvement of youth care. Study and sample characteristics moderated the effectiveness of FGC. The review is limited in scope as it only included families with older children who received a very specific service delivery model (Dijkstra et al., 2016).

Maltais et al. (2019) provide a systematic review and a series of meta‐analyses exploring the effects of interventions aimed at promoting parent engagement and family reunification for families with children in out‐of‐home care. Eight studies were included in the meta‐analyses. Each study examined the effectiveness of a goal‐oriented parental engagement intervention, relative to a control group made up of parents who received standard services. Results indicate that parents exposed to goal‐oriented engagement interventions showed greater engagement (effect size *d* = 0.71) and likelihood of reunification (effect size odds ratio [OR] = 2.49) than parents who received standard services. In particular, moderator analysis showed that parents who specifically participated in a family‐focused intervention showed the highest engagement in comparison to parents involved in other types of interventions or who received standard services (effect size *d* = 1.08). No moderators significantly explained the heterogeneity of studies on family reunification (Maltais et al., 2019). The review provides important knowledge, but is limited in scope, as it does not include interventions designed to prevent out‐of‐home placement in at‐risk families with no prior history of out‐of‐home placement.

Bezeczky et al. (2020) is a systematic review and meta‐analysis of the effectiveness of Intensive Family Preservation Services (IFPS) for families with children at imminent risk of out‐of‐home placement on measures of risk of out‐of‐home placement at the child and family level. Eligible IFPS interventions had to adopt the key service characteristics of the Homebuilders model. The review included 37 publications reporting on 33 studies. Results showed significant reductions in relative risk (RR) of out‐of‐home placements in children who received IFPS compared with controls at the child level at 3, 6, 12, and 24 months' follow‐up (Bezeczky et al., 2020). While this review contains important knowledge, the present review will include a much broader range of family preservation services and, if possible, a number of secondary outcomes as well as an exploration of the impact of potential moderators on measures of out‐of‐home placements.

In a Campbell systematic review, McGinn et al. (2020) assessed the effectiveness of the formal use of family group decision‐making (FGDM) on measures of child safety, permanence of child's living situation, and child and family well‐being. Eighteen eligible study reports were included reporting on 18 study samples. Four were RCTs. Ten effect sizes, from nine QESs, were synthesized to examine effects on family reunification or the effect on maintaining in‐home care; in short, the effect FGDM had on keeping families together. The overall effect was positive and statistically significant with an OR = 1.69 (confidence interval [1.03–2.78]). There was a high level of heterogeneity between the studies. The review summarizes evidence on a specific service delivery model for children who have been the subject of a child maltreatment investigation (McGinn et al., 2020). The present review will be broader in scope and include both a larger population and additional types of both preventative and reactive interventions for families with multiple types of risk factors.

LaBrenz et al. (2020) conducted a systematic review of interventions to reduce child welfare recidivism. The review included 10 studies exploring the effectiveness of interventions targeting successful reunification of children in care with their biological parents on measures of successful reunification or reunification that does not result in recidivism. Results were inconclusive, and the authors conclude that small sample sizes, lack of replication of studies, and small effect sizes limit the generalizability of findings (LaBrenz et al., 2020). The review provides important insights but is limited in scope as it does not include interventions to prevent out‐of‐home placement in at‐risk families with no prior history of out‐of‐home placement.

Littell et al. (2021) explored the effects of MST for out‐of‐home placement, social, emotional, and behavioral problems in youths aged 10 to 17 years old in a systematic review and meta‐analysis. The review included 23 trials, and the authors conclude that the quality of evidence for MST is mixed and effects are inconsistent across studies (Littell et al., 2021). While this review provides important insights, the present review will be broader in scope and include studies of interventions for families with children across the age span – including infants – and will include studies of interventions containing different treatment components than MST.

Neo et al. (2021) conducted a systematic review and meta‐analysis on the effects of integrated treatment programs for mothers with substance use problems on measures of out‐of‐home child placements. Six trials were included in the review, two RCTs and four non‐randomized controlled studies. The results showed that mothers who participated in integrated treatment programs were significantly less likely to have children removed from their care (OR = 0.40) (Neo et al., 2021). Although the review provides important knowledge, the scope of the review is narrow as it only included interventions for mothers with substance use problems, not fathers or parents with other risk factors or other types of family preservation interventions.

In addition to these more narrowly focused reviews, a number of systematic reviews with a broader focus are also available. These reviews need to be updated and in some cases, they do not contain a risk of bias assessment and/or they include studies without a control group, which limits conclusions regarding the effects of interventions.

In a meta‐analytic review, MacLeod and Nelson (2000) explored the effectiveness of programs in promoting family wellness and preventing child maltreatment (*k* = 56). The analyses used multiple types of outcomes relating to child maltreatment and family wellness including out‐of‐home placement. However, a separate meta‐analysis using only out‐of‐home placement outcomes was not performed.

Nelson et al. (2009) conducted a systematic review of family preservation research. The review does not contain a meta‐analysis, but reports individual effect sizes for included studies. Within the narrative synthesis, the authors point to a number of methodological problems and flaws within primary studies, but tentatively conclude that the effects of intensive family preservation efforts look “cautiously promising” (Nelson et al., 2009).

Al et al. (2012) conducted a meta‐analysis of intensive family preservation programs on measures of placement prevention and family functioning (*k* = 20). The results show that intensive family preservation programs had a positive effect on family functioning (*d* = 0.486), but were generally not effective in preventing out‐of‐home placement. The overall effect for prevention of out‐of‐home placement, which was based on 19 studies (*N* = 31,214), was not significant (*d* = 0.003) (Al et al., 2012). Although the review contains important knowledge, it needs to be updated and does not contain a risk of bias assessment of the included studies, which is where the present review will contribute.

Lee et al. (2014) conducted a systematic review aimed at identifying program and practice elements for placement prevention and their effectiveness (*k* = 37). The review included both RCTs, quasi‐experimental, and pre‐and post studies, and the authors did not perform a meta‐analysis. The review identified the most common clinical practice elements and these include: program monitoring, case management, accessibility promotion assessment and individual therapy for caregivers, interventions to promote problem‐solving skills, and family therapy. Effect size estimates for placement‐related outcomes (decreased out‐of‐home placement, decreased hospitalization, decreased incarceration, and decreased costs) were calculated to estimate the treatment effectiveness of the interventions and reported separately (Lee et al., 2014). This review needs to be updated and does not contain a risk of bias assessment of the included studies nor a meta‐analysis, which the present review will include.

van Assen et al. (2020) conducted a systematic review and meta‐analysis of home‐visiting interventions for families with complex and multiple problems with children aged 5–18 years old on measures of out‐of‐home placement rates and child outcomes. The review included 42 publications reporting on 50 studies. Most studies included in the review used a one‐group observational design (*k* = 40). A random‐effects survival curve meta‐analysis model was estimated for out‐of‐home placement and random‐effects meta‐analysis models were estimated for children's behavioral problems and stressful experiences. Out‐of‐home placement increased from 7.5% at case closure to 24.3% 1 year after case closure. On average, there was a moderate decrease in emotional and behavioral problems (*d* = 0.50) and stressful experiences (*d* = 0.50) during intervention, but considerable problems remained after case closure (van Assen et al., 2020). Although the review contributes with knowledge of relevant interventions, the scope of the review is narrow as it only included interventions for families with children aged 5 years old or above. Furthermore, the review only included interventions using specific home‐visitation models of family preservation interventions and the review included studies without a control or comparison group.

In summary, a number of previous systematic reviews have explored the effects of different types of specific family preservation/reunification and child abuse prevention interventions for specific populations, such as parents with substance abuse. Reviews with a broader focus are also available, but these need to be updated. Furthermore, these reviews mostly include studies without a control group or do not contain a risk of bias assessment of included studies and/or a meta‐analysis. Most reviews point to positive effects on a number of different outcomes including out‐of‐home placement. However, for the reasons discussed above, results are as of yet inconclusive.

As such, there is a need for a comprehensive and methodologically rigorous systematic review synthesizing evidence on the effects of all types of preventative interventions relating to out‐of‐home placement. We aim to fill this gap by performing extensive, up‐to‐date literature searches and applying state‐of‐the‐art meta‐analytic techniques to analyze the available evidence. It will also be a focus of the present review to analyze if some types of interventions are more effective than others by exploring potential moderators of the intervention effects. In sum, this review will provide updated knowledge of existing interventions and their effectiveness which will be useful to both local placement agencies and practitioners working within the field of child protection as well as to policy and decision makers with the power to decide on matters of child protection.

## 2 OBJECTIVES

The aim of the present review is to synthesize evidence on the effectiveness of interventions for at‐risk families aimed at preventing the out‐of‐home placement of children or increasing the likelihood that children are reunited with their birth families following temporary care arrangements. The review has two objectives:

1. To assess the effectiveness of interventions for at‐risk families with children aged between 0 and 17 years old on measures of out‐of‐home placement and on secondary outcomes.
2. To identify factors that modify intervention effectiveness (e.g., prior placements, parental risk factors such as substance abuse, mental health issues, age, minority status, child risk factors such as disabilities, age, and gender).

## 3 METHODS

### 3.1 Criteria for considering studies for this review

#### 3.1.1 Types of studies

To summarize what is known about the causal effects of preventative parenting interventions for at‐risk families with children aged 0–17 years old on measures of out‐of‐home placement, we will include studies with a well‐defined comparison group. The study designs eligible for inclusion are:

- RCTs
- QESs

There is no widely accepted definition of QES. For the purposes of this review, we will categoriese studies as QES based on the following criteria: (1) studies where the participants are allocated by actions controlled by the researcher (e.g., controlled trials); or (2) studies where the allocation to the intervention and control groups are not controlled by the researcher (e.g., by time differences or policy rules). Two main types of QES are designs that are able to account for unobservable sources of confounding and designs that adjust for observable confounders directly (Waddington et al., 2017). The former type include designs like the quasi‐random assignments of caseworkers to child maltreatment mentioned in Section 1, which use natural experiments or other mechanisms producing “as‐good‐as‐random” assignments to intervention and comparison groups (Bärnighausen et al., 2017). The latter type includes for example matching and regression approaches, in which the adjustment for confounders, in the best case, produces statistically equivalent intervention and comparison groups.

To be included, QESs must credibly demonstrate that outcome differences between intervention and comparison groups are the effect of the intervention and not the result of systematic baseline differences between groups. That is, selection bias should not be driving the results. This means that if the decision to place children in out‐of‐home care is based on an assessment of risk factors, the researchers need to rely on an mechanism that produces an as‐good‐as‐randomly assigned intervention and control group or be able to measure these risks and adjust for them. That is, if confounding is present, the researchers should use appropriate methods such as matching to ensure comparability between intervention and control groups at baseline. This assessment is included as part of the risk of bias tool, which we elaborate on in the Risk of bias section.

We include QES for two main reasons. First, placing a child in out‐of‐home care is a life‐altering decision. As the outcome entails very high stakes, randomly assigning interventions that influence the outcome may not be feasible, and in some contexts may neither be legal, nor ethical. Thus, the number of RCTs may be relatively small. Second, RCTs are often but not always and everywhere the study design with the highest internal validity (i.e., the lowest risk of bias; Sharma Waddington et al., 2022), and certainly not always the design with highest external validity (Degtiar & Rose, 2023; Bärnighausen et al., 2017). For example, in RCTs in the social sciences the individuals in the control group typically know that they are the control group (Sharma Waddington et al., 2022). This knowledge may affect their behavior (e.g., John Henry‐effects; Glennerster & Takavasha, 2013), which creates bias and threatens internal validity. Furthermore, RCTs frequently include a nonrepresentative subset of the population of interest (the target population) and it may be difficult to follow all participants from start to finish, which present challenges to external validity that QES may be better suited to tackle (Degtiar & Rose, 2023).

The comparison group can consist of no treatment, treatment as usual, other interventions/treatments offered, or wait‐list controls. In this review, comparison designs include studies in which more than one intervention is being investigated by the researcher, whereas treatment‐control designs include studies in which a specific intervention is compared to either no treatment or treatment as usual/normal service provison. Effect sizes from comparison studies in which two alternative interventions are compared against each other may not be fully comparable to effect sizes from treatment‐control designs. We therefore plan to analyze two‐treatment comparison designs separately from treatment‐control designs, or if data permits, use network meta‐analysis to combine the two types of studies (see Section 3.7.10 for more information). If two‐treatment comparison design effect sizes cannot be pooled, study‐level effects will be reported narratively. Within the meta‐analysis, the potential effects of different types of study designs on intervention effects will be explored using sensitivity analysis, which we describe in Section 3.7.10.

#### 3.1.2 Types of participants

We will include studies of at‐risk families (both single and two‐parent families) with at least one child aged between 0 and 17 years old at the beginning of the intervention. Parental risk factors will be defined broadly, and we will include all studies in which child protection services or other authorities have referred families for intervention. Further examples include studies in which parents have any of the following risk factors:

- Parental substance or alcohol abuse
- Teenage parenthood
- Poverty
- Criminality
- Domestic violence
- Previous placements of children in out‐of‐home care
- Parental mental health problems
- Parental physical health problems or disabilities
- Lack of social support

The above list is not exhaustive and we asume that most families – unless otherwise mentioned in the study – can be considered at risk if they are offered interventions aimed at reducing the risk of out‐of‐home placement. This means that if we come across studies of the effects of interventions offered to all families within a community – where most or all families are considered at risk – on measures of out‐of‐home placement, these studies will be included in the review.

#### 3.1.3 Types of interventions

Interventions included in this review may contain a number of different components such as case management, parental or child individual psychotherapy, MST, family therapy, housing assistance, 24‐h on‐call availability, weekend family homes, financial assistance, leisure activities, home consultant, in‐home services, family foster care or family institutional placement, support person, and mentoring (Lee et al., 2014; Storhaug & Kojan, 2017).

Eligible interventions must be aimed at preventing out‐of‐home placement of children and eligible studies must report on this primary outcome. This means that studies, which do not measure and evaluate the risk of out‐of‐home placement will be excluded, and that we will exclude studies of interventions aimed at decreasing parental risk factors, which do not report the effects on measures related to out‐of‐home placement.

#### 3.1.4 Types of outcome measures

##### Primary outcomes

The primary outcome in this review is out‐of‐home placement. That is, we aim to measure the extent to which interventions decrease the risk of children being placed in out‐of‐home care, decrease the number of days that children spend in temporary care arrangements, or increase the likelihood that children are reunited with their birth parents after being in temporary care arrangements.

The primary outcome may be measured as both a dichotomous variable and a continuous variable (number of days in care or time remaining with birth families). Outcomes may be measured by using administrative data, record reviews, or questionnaires filled out by child protection agencies or intervention participants.

##### Secondary outcomes

Secondary outcomes include: parent outcomes such as parenting stress, parental mental health and well‐being, child outcomes such as child mental health, educational attainment, attachment representations, behavior problems, well‐being, and parent/child relationship outcomes such as parental emotional availability and parental sensitivity. Studies containing only secondary outcomes will be excluded.

Secondary outcomes may be measured using standardized self‐reported questionnaires which have been used on other samples. For the child outcomes, questionnaires may be based on parent/caregiver/teacher report if the children are too young to self‐report. We will also include outcomes based on clinical interviews carried out by health care professionals.

#### 3.1.5 Duration of follow‐up

Time points for measures considered will be:

- Post‐intervention,
- Up to 1 year follow‐up,
- 1 to 2 year follow‐up,
- More than 2 year follow‐up,
- Follow‐up at any given point in time.

In summary, we will include follow‐up data regarding placement in out‐of‐home care during the remainder of the children's childhoods. Studies will only be included if they report on the primary outcome.

#### 3.1.6 Types of settings

To increase the comparability between institutional settings and the chances of obtaining results that can be transferred between settings, we require that families are residents in an OECD country. Studies from settings in countries outside the OECD will be excluded.

### 3.2 Search methods for identification of studies

Relevant published and unpublished studies will be identified through searches in electronic databases, gray literature repositories and resources, hand searches in specific targeted journals, citation‐tracking, contact to international experts, and Internet search engines.

If we come across errata these will also be included.

#### 3.2.1 Electronic searches

The following bibliographic databases will be searched:

– SocINDEX (EBSCO) 1908 – current
– PsycINFO (EBSCO) 1890 – current
– EconLit (EBSCO) 1969 – current
– ERIC (EBSCO) 1966 – current
– CINAHL (EBSCO) 1981 – current
– Academic Search Premier (EBSCO) 1931 – current
– Science Citation Index (Web of Science, Clarivate) 1900 – current
– Social Science Citation Index (Web of Science, Clarivate) 1956 – current
– Sociological Abstracts (ProQuest) 1952 – current

##### Description of search string

For the electronic searches, we will use a seach string based on the PICO(s)‐model, containing terms for the population and intervention. In designing the search string, we have drawn on our knowledge about content matter terminology and information retrieval methods and have also taken inspiration from the search terms used in previous reviews. Below are two examples of search strategies in databases with and without a thesaurus, respectively. Whenever adequate and possible, the conducted searches will include subject heading searches from the thesauri or indexes of the respective databases.

### 3.3 Examples of search strings

#### 3.3.1 Example of search strategy in a database with a thesaurus

**APA PsycINFO (EBSCO) 1890 – current**

Expanders – Apply equivalent subjects

Search modes – Boolean/Phrase

Searched 30.01.2024

**Table:** 

#	Query	Results
S13	S4 AND S8 AND S12	1989
S12	S9 OR S10 OR S11	32,724
S11	DE “Family Reunification” OR DE “Family Preservation” OR (ZU “family preservation”) OR (ZU “family reunification”)	645
S10	AB placement* OR “placement stabilit*” OR “family preserv*” OR “family reunifi*” OR “out‐of‐home placement*” OR “out of home placement*” OR “out‐of‐home care*” OR “out of home care*”	30,974
S9	TI placement* OR “placement stabilit*” OR “family preserv*” OR “family reunifi*” OR “out‐of‐home placement*” OR “out of home placement*” OR “out‐of‐home care*” OR “out of home care*”	6616
S8	S5 OR S6 OR S7	523,827
S7	(DE “Home Visiting Programs” OR DE “Family Preservation” OR DE “Family Therapy”) OR (DE “Family Intervention”) OR (DE “Family Reunification” OR DE “Foster Care” OR DE “Protective Services”) OR (ZU “family intervention”) or (ZU “family preservation”) or (ZU “family therapy”) or ((ZU “family reunification”) or (ZU “family support”) or ((ZU “child protective services”) or (ZU “foster home care”)	43,752
S6	AB “child protective service*” OR “home visit*” OR “family support program*” OR “therapeut* visit*” OR intervention* OR “in‐home intervention*” OR “in home intervention*” OR “family preservation service*” OR “in‐home therap*” OR “in home therap*” OR “in‐home family‐focused reunification*” OR “in home family‐focused reunification*” OR “family reunification*” OR “foster care” OR “home‐based services” OR “homebased services” OR “in‐home services” OR “family centred” OR “family centered”	486,463
S5	TI “child protective service*” OR “home visit*” OR “family support program*” OR “therapeut* visit*” OR intervention* OR “in‐home intervention*” OR “in home intervention*” OR “family preservation service*” OR “in‐home therap*” OR “in home therap*” OR “in‐home family‐focused reunification*” OR “in home family‐focused reunification*” OR “family reunification*” OR “foster care” OR “home‐based services” OR “homebased services” OR “in‐home services” OR “family centred” OR “family centered”	96,169
S4	S1 OR S2 OR S3	212,223
S3	(MM “Parents” OR DE “Adolescent Pregnancy” OR DE “Adolescent Fathers” OR DE “Adolescent Mothers” OR DE “Family Conflict” OR DE “Domestic Violence” OR DE “Dysfunctional Family”) OR (ZU “family conflict”) OR (ZU “family crises”)	54,967
S2	AB (parent* OR family* OR families) AND AB (“risk factor*” OR “at‐risk” OR “at risk” OR “substance* abus*” OR “alcohol abus*” OR “drug abus*” OR “child abus*'” OR “mental health problem*” or “mental illness*” or “mental disorder*” or “psychiatric illness*” OR “physical health problem*” OR disabilit* OR poverty or “low‐income” or “low socioeconomic” or “disadvantage*” OR poor OR criminal* OR “domestic violence” or “domestic abuse” OR “family violence” OR teenage* OR “multi problem famil*” OR “multi‐problem famil*” OR “multi‐stressed famil*” OR “multi‐challenged famil*” OR “vulnerable famil*” OR “troubled famil*” OR “complex famil*” OR “multiple risk* famil*” OR depriv* OR “multiple needs famil*”)	170,023
S1	TI (parent* OR family* OR families) AND TI (“risk factor*” OR “at‐risk” OR “at risk” OR “substance* abus*” OR “alcohol abus*” OR “drug abus*” OR “child abus*'” OR “mental health problem*” or “mental illness*” or “mental disorder*” or “psychiatric illness*” OR “physical health problem*” OR disabilit* OR poverty or “low‐income” or “low socioeconomic” or “disadvantage*” OR poor OR criminal* OR “domestic violence” or “domestic abuse” OR “family violence” OR teenage* OR “multi problem famil*” OR “multi‐problem famil*” OR “multi‐stressed famil*” OR “multi‐challenged famil*” OR “vulnerable famil*” OR “troubled famil*” OR “complex famil*” OR “multiple risk* famil*” OR depriv* OR “multiple needs famil*”)	17,084

#### 3.3.2 Example of search strategy in a database without a thesaurus

**SocINDEX (EBSCO) 1908 – current**

Expanders – Apply equivalent subjects

Search modes – Boolean/Phrase

Searched 30.01.2024

**Table:** 

#	Query
S13	S4 AND S8 AND S12
S12	S9 OR S10 OR S11
S11	(DE “FAMILY reunification”) OR (ZW “out‐of‐home placement”) or (ZW “out‐of‐home placements”)
S10	AB placement* OR “placement stabilit*” OR “family preserv*” OR “family reunifi*” OR “out‐of‐home placement*” OR “out of home placement*” OR “out‐of‐home care*” OR “out of home care*”
S9	TI placement* OR “placement stabilit*” OR “family preserv*” OR “family reunifi*” OR “out‐of‐home placement*” OR “out of home placement*” OR “out‐of‐home care*” OR “out of home care*”
S8	S5 OR S6 OR S7
S7	(DE “CHILD protection services” OR DE “FAMILY support” OR DE “STRUCTURAL family therapy” OR DE “FAMILY psychotherapy”) OR (DE “FAMILY reunification services” OR DE “FAMILY reunification”) OR (DE “FOSTER home care”) OR (ZW “family services, counseling, and therapy”) OR (ZW “therapeutic foster care”) or (ZW “in‐home family therapy”) or (ZW “home‐based family therapy”)
S6	AB “child protective service*” OR “home visit*” OR “family support program*” OR “therapeut* visit*” OR intervention* OR “in‐home intervention*” OR “in home intervention*” OR “family preservation service*” OR “in‐home therap*” OR “in home therap*” OR “in‐home family‐focused reunification*” OR “in home family‐focused reunification*” OR “family reunification*” OR “foster care” OR “home‐based services” OR “homebased services” OR “in‐home services” OR “family centred” OR “family centered”
S5	TI “child protective service*” OR “home visit*” OR “family support program*” OR “therapeut* visit*” OR intervention* OR “in‐home intervention*” OR “in home intervention*” OR “family preservation service*” OR “in‐home therap*” OR “in home therap*” OR “in‐home family‐focused reunification*” OR “in home family‐focused reunification*” OR “family reunification*” OR “foster care” OR “home‐based services” OR “homebased services” OR “in‐home services” OR “family centred” OR “family centered”
S4	S1 OR S2 OR S3
S3	DE “ABUSIVE parents” OR DE “FAMILY conflict” OR DE “DOMESTIC violence” OR DE “ALCOHOL & parents” OR DE “TEENAGE fathers” OR DE “TEENAGE parents” OR DE “TEENAGE mothers” OR DE “LOW‐income parents” OR (ZW “multi‐problem families”) OR (ZW “multi‐problem family”)
S2	AB (parent* OR family* OR families) AND AB (“risk factor*” OR “at‐risk” OR “at risk” OR “substance* abus*” OR “alcohol abus*” OR “drug abus*” OR “child abus*'” OR “mental health problem*” or “mental illness*” or “mental disorder*” or “psychiatric illness*” OR “physical health problem*” OR disabilit* OR poverty or “low‐income” or “low socioeconomic” or “disadvantage*” OR poor OR criminal* OR “domestic violence” or “domestic abuse” OR “family violence” OR teenage* OR “multi problem famil*” OR “multi‐problem famil*” OR “multi‐stressed famil*” OR “multi‐challenged famil*” OR “vulnerable famil*” OR “troubled famil*” OR “complex famil*” OR “multiple risk* famil*” OR depriv* OR “multiple needs famil*”)
S1	TI (parent* OR family* OR families) AND TI (“risk factor*” OR “at‐risk” OR “at risk” OR “substance* abus*” OR “alcohol abus*” OR “drug abus*” OR “child abus*'” OR “mental health problem*” or “mental illness*” or “mental disorder*” or “psychiatric illness*” OR “physical health problem*” OR disabilit* OR poverty or “low‐income” or “low socioeconomic” or “disadvantage*” OR poor OR criminal* OR “domestic violence” or “domestic abuse” OR “family violence” OR teenage* OR “multi problem famil*” OR “multi‐problem famil*” OR “multi‐stressed famil*” OR “multi‐challenged famil*” OR “vulnerable famil*” OR “troubled famil*” OR “complex famil*” OR “multiple risk* famil*” OR depriv* OR “multiple needs famil*”)

#### 3.3.3 Limitations of the search string

We will not implement any restrictions to our searches based on publication date or language. In screening and processing the references found, we will however be limited by the language proficiencies available in the review team which allow us to consider studies published in English, Danish, Norwegian, and Swedish. In accordance with this, specific gray literature searches will be performed to locate studies written in Scandinavian languages.

### 3.4 Searching other resources

We will search specifically after four types of gray literature: working papers, reports, dissertations, and conference proceedings. We had planned to perform seaches in ProQuest Dissertations & Theses Global (ProQuest), Conference Proceedings Citation Index, and Index of Conference Proceedings, but are unable to do so due to lack of access. We believe nonetheless that our other searches will be comprehensive enough to secure adequate coverage of both dissertations and conference proceedings. Worth noting is also that several of the databases included in the literature search (Academic Search Premier, CINAHL, Science Citation Index Expanded [Web Of Science], and Social Sciences Citation Index [Web Of Science]) already include conference proceedings. Some of the bibliographic databases also cover gray literature (e.g., ERIC).

We will search the following resources for gray literature:

– EBSCO Open Dissertations (dissertations) (EBSCO‐host) – https://biblioboard.com/opendissertations/
– Google Scholar (reports, working papers, dissertations) – https://scholar.google.com/
– Google searches (reports, working papers, dissertations) – https://www.google.com/
– Social Care Online (reports, working papers, dissertations, systematic reviews) – https://www.scie-socialcareonline.org.uk/ (only updated until January 2023)
– Social Science Research Network (working papers) – https://www.ssrn.com/index.cfm/en/
– OECD iLibrary (working papers, conference proceedings) – https://www.oecd-ilibrary.org/
– NBER working paper series – http://www.nber.org
– CORE – research outputs from international repositories – https://core.ac.uk/
– SocArXiv – open archive of the social sciences (working papers, preprints, and published papers) – https://socopen.org/
– Abt Associates (reports) – https://www.abtassociates.com/
– American Institutes for Research (reports) – https://www.air.org/
– The Title IV‐E Prevention Services Clearinghouse – https://preventionservices.acf.hhs.gov/
– Danish National Research Database (working papers, articles, dissertations, systematic reviews) – https://forskningsportal.dk
– NORA – Norwegian Open Research Archives – http://nora.openaccess.no/
– SwePub – Academic publications at Swedish universities – http://swepub.kb.se/
– DIVA – Swedish Digital Scientific Archives – http://www.diva-portal.org/smash/

Further resources for identifying gray literature may be added during the search process. When selecting gray literature resources, we have consulted the list of websites comprised in the article by Kugley 2017 (https://revman.cochrane.org/#/111220060308495667/dashboard/htmlView/1.3#REF-Kugley-2017_x00a0_). A final list of gray literature resources will be included in the appendix of the review.

### 3.5 Hand search

We will conduct hand searches of selected journals to make sure that all relevant articles are found. The following eight journals have been selected on the basis of our initial test searches where they included the most relevant articles:

- Children and Youth Services Review
- Child Maltreatment
- Child Abuse and Neglect
- Research on Social Work Practice
- Journal of Child and Family Studies
- Child and Family Social Work
- Journal of Public Child Welfare
- Adoption & Fostering

The hand searches will focus on editions published from 2021 and up to the present time to secure recent unpublished articles which have not yet been indexed in the bibliographic databases.

### 3.6 Search for systematic reviews

As part of our preparations for this protocol, we developed a specific search string to identify other systematic reviews relevant to our review question and applied it to two of the databases judged most likely to cover the subject matter: PsycINFO (EBSCO) and SocINDEX (EBSCO). This was done simultaneous with the development of the search string described above. The identified relevant reviews were used to develop our search terms and are considered in this protocol in Section 1 (under the heading “Why it is important to do this review”). During the review process, we will search for further systematic reviews in the following resources:

- Campbell Systematic Reviews – https://campbellcollaboration.org/
- Cochrane Library – https://www.cochranelibrary.com/
- Center for Reviews and Dissemination Databases – https://www.crd.york.ac.uk/CRDWeb/
- EPPI‐Center Systematic Reviews – Database of Education Research – https://eppi.ioe.ac.uk/cms/Databases/tabid/185/Default.aspx

If we locate other relevant systematic reviews, we will consider them in the full review and use them for citation‐tracking (see next section).

#### 3.6.1 Citation tracking

To identify both published and unpublished studies that may not have been caught in our main searches, we will utilize citation‐tracking/snowballing strategies. Our primary strategy will be to citation‐track related systematic reviews and meta‐analyses, as well as included primary studies, using both forward and backward citation‐tracking.

#### 3.6.2 Contact with international experts

We will contact international experts to identify potentially relevant studies not already located through our other searches. This will be done by providing the experts with the inclusion criteria for the review along with the list of included studies, asking for any other published or unpublished studies relevant to the review. We expect to primarily contact corresponding authors of related reviews, but contacts will also be extended to others if we find references to or mentions of relevant studies in screened publications.

### 3.7 Data collection and analysis

#### 3.7.1 Description of methods used in primary research

We expect to include both randomized studies and non‐randomized studies.

To be eligible for inclusion, studies comparing two groups of families/children must adequately deal with between‐group differences on relevant variables at baseline (e.g., mental health, socioeconomic background, ethnicity, and alcohol/drug abuse). We will assess the methodological appropriateness according to the risk of bias model outlined in Section 3.7.4.

An example of an eligible study for the present review is Prinz et al. (2009). The study explored the effectiveness of the Triple P—Positive Parenting Program system, which is a multicomponent manualized intervention consisting of five intervention levels of increasing intensity and narrowing population reach. In the study, 18 counties in the United States were randomly assigned to either dissemination of the Triple P—Positive Parenting Program system or to the services‐as‐usual control condition. Outcomes include records of child out‐of‐home placements obtained through the foster care system.

#### 3.7.2 Selection of studies

Under the supervision of review authors, two review team assistants will first independently screen titles and abstracts to exclude studies that are clearly irrelevant. Studies considered eligible by at least one assistant or studies where there is insufficient information in the title and abstract to judge eligibility will be retrieved in full text. The full texts will then be screened independently by two review team assistants under the supervision of the review authors. Any disagreement of eligibility will be resolved by the review authors. Screening on both title/abstract and full text will be performed using EPPI Reviewer 6 software (Thomas et al., 2023). Exclusion of studies that otherwise might be expected to be eligible will be documented and presented in an appendix.

The study inclusion criteria will be piloted by the review authors. The overall search and screening process will be illustrated in a flow diagram. None of the review authors will be blind to the authors, institutions, or journals responsible for the publication of articles.

#### 3.7.3 Data extraction and management

Two review team members will independently code and extract data from included studies. A coding sheet will be piloted on several studies and revised as necessary.

Disagreements will be resolved by consulting a third review team member. Disagreements resolved by a third review team member will be reported. Data and information will be extracted on available characteristics of participants, intervention characteristics and control conditions, research design, sample size, risk of bias and potential confounding factors, outcomes, and results. Extracted data will be stored electronically.

#### 3.7.4 Assessment of risk of bias in included studies

We will assess the risk of bias in randomized studies using Cochrane's revised risk of bias tool, RoB 2 (Higgins et al., 2019). The tool is structured into five domains, each with a set of signaling questions to be answered for a specific outcome. The five domains cover all types of bias that can affect the results of randomized trials. The five domains for individually randomized trials are:

- bias arising from the randomization process;
- bias due to deviations from intended interventions;
- bias due to missing outcome data;
- bias in measurement of the outcome;
- bias in selection of the reported results.

If we include cluster‐randomized trials, an additional domain is included: (1b) Bias arising from identification or recruitment of individual participants within clusters. We will use the latest template for completion (currently it is the version of August 22, 2019, for individually andomized parallel‐group trials and March 18, 2021, for cluster‐randomized parallel‐group trials).

We will assess the risk of bias in non‐randomized studies using the model ROBINS‐I, developed by members of the Cochrane Bias Methods Group and the Cochrane Non‐Randomized Studies Methods Group (Sterne, Hernán, et al., 2016). We will use the latest template for completion (currently it is the version of September 19, 2016).

The ROBINS‐I tool covers seven domains (each with a set of signaling questions to be answered for a specific outcome) through which bias might be introduced into non‐randomized studies:

- bias due to confounding;
- bias in selection of participants;
- bias in classification of interventions;
- bias due to deviations from intended interventions;
- bias due to missing outcome data;
- bias in measurement of the outcome;
- bias in selection of the reported results.

The first two domains address issues before the start of the interventions and the third domain addresses classification of the interventions themselves. The last four domains address issues after the start of interventions and there is substantial overlap for these four domains between bias in randomized studies and bias in non‐randomized studies (although signaling questions are somewhat different in several places, see Higgins et al., 2019; Sterne, Higgins, et al., 2016).

Randomized study outcomes are rated on a “Low/Some concerns/High” scale on each domain, whereas non‐randomized study outcomes are rated on a “Low/Moderate/Serious/Critical/No Information” scale on each domain. The level “Critical” means that the study (outcome) is too problematic in this domain to provide any useful evidence on the effects of the intervention and it is excluded from the data synthesis. We will stop the assessment of a non‐randomized study outcome as soon as one domain in the ROBINS‐I is judged as “Critical.” The same critical level of risk of bias (excluding the result from the data synthesis) is not present in the RoB 2 tool (Higgins et al., 2019).

In both tools, an overall rating may be made on the basis of the domain ratings. A rating of “some concerns” in multiple domains of RoB 2 may lead to a decision of an overall judgment of “high” risk of bias for that outcome. A “serious” risk of bias in multiple domains of ROBINS‐I may lead to a decision of an overall judgment of “critical” risk of bias for that outcome, and it will be excluded from the data synthesis. Outcome measures which have been excluded due to multiple ratings of “serious” in individual domains will be listed in the final review, along with reasons for the exclusions.

The overall rating of the study also contains an assessment of the overall bias direction for the assessed outcomes in both tools. A further commonality is that both tools require pre‐specification of the effect type that will be assessed (see Section 3.7.4.2).

In the case of an RCT, where there is evidence that the randomization has gone wrong or is no longer valid, we will assess the risk of bias of the outcome measures using ROBINS‐I instead of RoB 2. Examples of reasons for assessing RCTs as non‐randomized studies may include studies showing large and systematic differences between treatment conditions while not explaining the randomization procedure adequately; studies with large‐scale differential attrition between conditions in the sample used to estimate the effects; or studies selectively reporting results for some part of the sample or for only some of the measured outcomes. In such cases, differences between the treatment and control conditions are likely systematically related to other factors than the intervention and the random assignment is, on its own, unlikely to produce unbiased estimates of the intervention effects. As ROBINS‐I allows for an assessment of for example confounding, we believe it is more appropriate to assess effect sizes from studies with invalid randomization using ROBINS‐I than RoB 2. If so, we will report this decision as part of the risk of bias assessment of the outcome measure in question. As other effect sizes assessed with ROBINS‐I, these effect sizes may receive a “Critical” rating and be excluded from the data synthesis.

##### Confounding and importance of prespecified factors

An important part of the risk of bias assessment of non‐randomized studies is consideration of how the studies deal with confounding factors. Systematic baseline differences between groups can compromise comparability between groups. Baseline differences can be observable (e.g., age and gender) and unobservable to the researcher(e.g., parental motivation and “ability”). There is no single non‐randomized study design that always solves the selection problem. Different designs represent different approaches to dealing with selection problems under different assumptions, and consequently require different types of data. There can be particularly great variations in how different designs deal with selection on unobservables. The “adequate” method depends on the model generating participation, that is, assumptions about the nature of the process by which participants are selected into a program.

A major difficulty in estimating causal effects of parenting interventions is the potential heterogeneity of both the different child protection legislation and institutional settings and the diversity of parental and child risk factors. There may be unobservable factors affecting parenting ability and child development or invisible selection mechanisms causing certain types of families to avoid contact with authorities for reasons unavailable to the researcher.

As there is no universally correct way to construct counterfactuals for non‐randomized designs, we will look for evidence that identification is achieved, and that the authors of the primary studies justify their choice of method in a convincing manner by discussing the assumption(s) leading to identification of the assumption(s) that make it possible to identify the counterfactual. Preferably the authors should make an effort to justify their choice of method and convince the reader that the at‐risk families, children, and settings in each study condition are comparable.

ROBINS‐I dictates that reviewers should define critical confounders relevant to most or all eligible studies at the protocol stage. In addition to unobservable confounding factors, we believe that it will be important to assess the following parental risk factors: mental health, socioeconomic background, ethnicity, and alcohol/drug abuse as the most important potential confounding factors. In each study, we will assess whether these factors have been considered. If other confounders are unbalanced between the intervention and control group, or between the comparison groups, the lack of balance may be reflected in a higher rating (i.e., defining critical confounders does not imply that other confounders will not be considered).

##### Effect of primary interest and important co‐interventions

We are interested in both the effect of assignment to intervention, that is, the intention to treat (ITT) effect, and the effect of participating in and completing the intended intervention, that is, the treatment on the treated (TOT) effect. The risk of bias assessments will therefore be in relation to both effects, whenever both types of effect estimates are available. We will analyze ITT and TOT effect sizes separately (see Section 3.7.10 for more information).

The risk of bias assessments of both randomized trials and non‐randomized studies will consider adherence and differences in additional interventions (“co‐interventions”) between intervention groups. Important co‐interventions will be the regular support systems available to families at risk.

##### Assessment

At least two review authors will independently assess the risk of bias for each relevant outcome from the included studies. Any disagreements will be resolved by a third reviewer with content and statistical expertise. We will report the risk of bias assessment in risk of bias tables for each included study outcome in the completed review.

#### 3.7.5 Measures of treatment effect

##### Continuous outcomes

For continuous outcomes, effect sizes with 95% confidence intervals will be calculated, where means and standard deviations are available. If means and standard deviations are not available, we will calculate standardized mean differences (SMDs) from *F*‐ratios, *t*‐values, *χ* ^2^ values, and correlation coefficients, where available, using the methods suggested by Wilson and Lipsey (2001). If insufficient information is yielded, the review authors will request this information from the principal investigators. Hedges' *g* will be used for estimating SMDs (Hedges, 1981). Some outcome variables may be reported on scales that can be directly compared across studies. In this case, we will not convert the variable to *g*. Time remaining with birth family/time in out‐of‐home care is an example of a primary outcome measure that may be reported on a scale (e.g., days or months) that may be comparable across studies. Any standardized measure of child psychosocial adjustment and mental health are examples of relevant secondary continuous outcomes in this review, which likely will have to be converted to *g*.

##### Dichotomous outcomes

For dichotomous outcomes, we will calculate ORs with 95% confidence intervals. Placement in out‐of‐home care or not may be an example of a dichotomous outcome in this review.

There are statistical approaches available to re‐express dichotomous and continuous data, which enable pooling the two types of outcomes in the analysis (Sánchez‐Meca et al., 2003). To calculate a common metric, ORs will be converted to SMD effect sizes using the Cox transformation, or vice versa, depending on whichever is the most common outcome type. We will only transform dichotomous effect sizes to SMD if appropriate, as may be the case with for example the outcome attachment security that can be measured with both binary and continuous data. When effect sizes cannot be pooled, study‐level effects will be reported in as much detail as possible. Software for storing data and statistical analyses will be Excel and R.

#### 3.7.6 Criteria for determination of independent findings

We will take into account the unit of analysis of the studies to determine whether individuals/families were randomized in groups (i.e., cluster‐randomized trials), whether individuals/families may have undergone multiple interventions, whether there were multiple treatment groups, and whether several studies are based on the same data source.

##### Cluster‐assigned treatment

The assignment of treatment in clusters can result in an overestimation of the precision of the results (with a higher risk of a Type I error) if the clustering is not accounted for in the analysis. If we include studies using cluster‐assignment, the impact of the inclusion of data from such studies in the meta‐analyses will be explored using a sensitivity analysis and any necessary adjustments to the data will be made, using estimates of the intra‐cluster correlation (ICC), if available in the studies. If study‐level ICCs are not available, we will adjust effect sizes using a common ICC for all studies with cluster‐assigned treatment that lack information. We will use the median of reported ICCs in studies that report the ICCs to adjust effect sizes in studies that lack this information.

##### Multiple interventions groups and multiple interventions per individuals

We are unlikely to identify cross‐over studies as the effects of therapy are intended to be long term. Therefore, cross‐over from a treatment condition to no‐treatment condition would not be feasible. For studies with more than one active intervention and only one control group, we will include all interventions and account for the dependent effect sizes as described in Section 3.7.10.

##### Multiple studies using the same sample of data

In some cases, several studies may have used the same sample of data or some studies may have used only a subset of a sample used in another study. We will review all such studies, but in the meta‐analysis, we will only include one estimate of the effect for each conceptual outcome from each sample of data. This is done to avoid dependencies between the “observations” (i.e., the estimates of the effect) in the meta‐analysis. The choice of which estimate(s) to include will be based on our risk of bias assessment of the studies. If there are multiple estimates of effects regarding the same outcome (such as child mental health), we will choose the estimate from the study that we judge to have the least risk of bias (primarily, confounding bias). If two (or more) studies are judged to have the same risk of bias and one (or more) of the studies uses a subset of a sample used in another study (or studies), we will include the study using the full set of participants.

##### Multiple time points

When the results are measured at multiple time points, each outcome at each time point will be analyzed in a separate meta‐analysis with other comparable studies taking measurements at a similar time point. As a general guideline, these will be grouped together as follows: (a) post‐intervention, (b) up to 1 year follow up, (c) 1 to 2 year follow up, and (d) More than 2 year follow up. However, should the studies provide viable reasons for an adjusted choice of relevant and meaningful duration intervals for the analysis of outcomes, we will adjust the grouping.

#### 3.7.7 Dealing with missing data

Missing data in the individual studies will be assessed using the risk of bias tool. Studies must permit calculation of a numeric effect size for the outcomes to be eligible for inclusion in the meta‐analysis. Where studies have missing summary data, such as missing standard deviations, we will derive these where possible from, for example, *F*‐ratios, *t*‐values, *χ* ^2^ values, and correlation coefficients using the methods suggested by Wilson and Lipsey (2001). If these statistics are also missing, the review authors will request information from the study investigators.

If missing summary data necessary for the calculation of effect sizes cannot be derived or retrieved, the study results will be reported in as much detail as possible, that is, the study will be included in the review but excluded from the meta‐analysis.

#### 3.7.8 Assessment of heterogeneity

Heterogeneity can stem from either an expected variation in effects or from sampling errors in included studies. In this review, we assume that variation in effects will occur across contexts and studies and will therefore use a random‐effects model in our main analysis (see also Section 3.7.10). We will calculate and report the within‐study standard deviation (*ω*), the between‐study standard deviation (*τ*), the total standard deviation (*σ* = *√*(*ω* ^2^ + *τ* ^2^)), *Q* tests, *I*² statistics, and prediction intervals to assess heterogenity.

#### 3.7.9 Assessment of reporting biases

Reporting bias might refer to both publication bias and selective reporting of outcome data and results. Bias from selective reporting of outcome data and results are assessed in both RoB 2 and ROBINS‐I.

We intend to use the following methods to assess the extent of publication bias. First, we will show funnel plots and examine whether they are asymmetric (Higgins et al., 2011). To formally test for asymmetry, we will use a version of Egger's test (Egger et al., 1997) suggested by Rodgers and Pustejovsky (2021). Pustejovsky and Rodgers (2019) showed that the original Egger's test rejected the null hypothesis of no asymmetry at higher rates than the chosen level of statistical significance (i.e., the Type I errors were inflated). In the simulations reported in Rodgers and Pustejovsky (2021), their “Egger Sandwich” test, which takes dependence between effect sizes into account, had better properties in terms of Type I errors than the original Egger's test, and other tested methods. As Rodgers and Pustejovsky (2021), we will interpret the rejection of the null hypothesis of no asymmetry in a one‐sided test with significance level 0.05 as an indication of asymmetry.

Asymmetry is not necessarily caused by publication bias (and publication bias does not necessarily cause asymmetry). If asymmetry is present, we will consider possible reasons for this pattern and test how sensitive our results are to publication bias using the method developed by Mathur and VanderWeele (2020). Furthermore, Egger's test, in both the regular and “Sandwich” version, has a limited capacity to detect publication bias when the number of included studies is small (Eggers et al., 1997; Rodgers & Pustejovsky, 2021), which may be the case in our review. We will also, if the number of studies permit it, consider using selection models (e.g., Andrews & Kasy, 2019; Hedges, 1992; Hedges & Vevea, 2005), which may identify and correct for the presence of publication bias.

#### 3.7.10 Data synthesis

Our planned data synthesis has the following steps: First, we will provide descriptive summaries of the participant, contextual, methodological, and outcome characteristics for the studies included in the data synthesis.

Second, depending on whether all out‐of‐home placement outcomes can be pooled and the type of effect estimates, our main effects analysis will report one or more weighted average effect sizes comparing the out‐of‐home placement of children in the the intervention groups and the control groups (corresponding to our first objective). We will present forest plots and heterogeneity measures. We will analyze ITT and TOT estimates separately. Studies of interventions for families in which the child has never been in out‐of‐home placement will be analyzed seperately from family reunification interventions. However, in some studies of preventive interventions some of the participating children may be taken into emergency custody during the study period. If studies include families where children have spent time in temporary care arrangements during the study period, and if outcomes include measures such as time spent in care or time spentd with the birth family, we may decide to include these in a meta‐analysis of family reunification interventions.

Third, as far as our data permit, we will conduct moderator analyses (described in Section 3.7.11) and analyses of secondary outcomes (corresponding to our second objective). We intend to perform all statistical analyses in R.

As we expect heterogeneity across contexts and studies, we assume a random‐effects model in all our meta‐analyses. We will use inverse‐variance based weights and the correlated‐hierarchical effects (CHE‐RVE) model developed by Pustejovsky and Tipton (2022) in the main effects and moderator analysis. This model will allow us to take into account multi‐level structures of the data, with effect sizes nested in treatments and studies, and dependencies between effect sizes that arise because there are more than one outcome reported for the same sample (“correlated effects”) and because multiple, nonoverlapping samples are included in the same study (“hierarchical effects”). Both these types of dependencies are conceivable in our case. Further advantages of the CHE‐RVE model is that it uses the restricted maximum likelihood (REML) estimator included in the R package metafor (Viechtbauer, 2022) to estimate the variance components, and robust variance estimation (RVE) to estimate the standard errors and confidence intervals. Both REML and RVE performs well and compare favorably to other methods in simulations (Fernandez‐Castilla et al., 2021; Langan et al., 2019; Vembye et al., 2023). The CHE‐RVE model is implemented in three steps:

In Step 1, we identify an appropriate working model based on the features of our sample (e.g., whether there are correlated or hierarchical effects, or both). The CHE‐RVE model requires a baseline value for the correlation between pairs of effect sizes from the same study (*ρ*). As Pustejovsky and Tipton (2022), we will use *ρ* = 0.6 in our primary analyses and test for the sensitivity of higher and lower values.

In Step 2, we will estimate meta‐regressions using a combination of the *clubSandwich* (Pustejovsky, 2022) and *metafor* (Viechtbauer, 2022) packages in R. The meta‐regressions will be estimated separately for ITT and TOT estimates, and by measurement timing (as described in Section 3.7.6.4). For the main effects analysis, we will regress the dependent variables (primary and secondary outcomes) on an intercept only, which provides the weighted average effect size. As described in the next section, the moderator analysis will add explanatory variables to this specification. We will use the *clubSandwich* package to specify the correlation structure between effect size estimates within studies, and estimate the random‐effects variance components, inverse‐variance weight matrices, and the meta‐regression coefficients using the REML procedure in the *metafor* package.

In Step 3, we will calculate confidence intervals based on the RVE standard errors obtained from the *clubSandwich* package. The confidence intervals will be based on standard errors, which are adjusted for small‐sample bias as suggested by Tipton (2015) and Tipton and Pustejovsky (2015). We will use the CR2 adjustment. We will report 95% confidence intervals and the small‐sample adjusted degrees of freedom for all analyses.

A note is warranted about the interpretation of the analysis of secondary outcomes. Because we require studies to have measured effects on out‐of‐home placement to be included and many otherwise relevant studies may not include this outcome, the included interventions in this review are unlikely to be representative of all interventions that target families at risk of out‐of‐home placements. Therefore, the effects we will estimate on the secondary outcomes are similarly unlikely to be representative. Our purpose with the analysis of secondary outcomes is instead to enable a more comprehensive evaluation of the interventions and identify factors that may explain why some interventions are more effective than others. For example, interventions that effectively prevent out‐of‐home care may affect child well‐being negatively, and be harmful on balance. Moreover, if interventions targeting parental stress do not reduce the risk of out‐of‐home placement, a reason may be that the interventions did not reduce parental stress as intended. That is, by examining secondary outcomes, we may find indications of whether the interventions worked as intended or not, and thereby find explanations of heterogeneity or lack of effects.

#### 3.7.11 Subgroup analysis and investigation of heterogeneity

There are many factors that may explain the potential heterogeneity of effect sizes: study design, participant characteristics (e.g., studies considering a specific population such as parents with drug abuse, teenage mothers, or children with disabilities), the duration and intensity of the intervention, and the specific components included in the intervention or the type of intervention. The latter is of particular interest in this review, which will likely include many intervention types. A salient question is therefore if some intervention components or types have larger effects than others. Although this question is very interesting, we do not know what interventions we will include and can therefore not prespecify the analysis. Instead, we will conduct exploratory moderator analyses of intervention components/types (see further below).

Moderator and sub‐group analysis in previous reviews have found moderating effects of study design and risk factors (e.g., Al et al., 2012; Dijkstra et al., 2016; Macleod & Nelson, 2000; Maltais et al., 2019). However, except for study design, no moderator was significantly correlated with the effect sizes in more than one moderator analysis. In our confirmatory moderator analysis, we will start by testing whether RCTs and QES have similar effects by including an indicator for QES in a meta‐regression. Should we find large differences that cannot be explained by other features of the interventions, we will consider analyzing RCTs and QES separately.

Regarding other moderators, several analyses used single‐factor subgroup analysis, which, as moderators are often associated, makes it difficult to learn the moderating effects of each moderator, conditional on the others. Thus, it is difficult to use earlier meta‐analyses to prioritize further among moderators. If the number of included studies is sufficient and given that there is variation in the covariates, we will perform moderator analyses by including more explanatory variables (moderators) in the meta‐regression framework described in the previous section to explore how the moderators are related to heterogeneity. As moderators may be correlated, we prefer to include all variables in one meta‐regression. However, as it decreases the degrees of freedom, adding all moderators simultaneously may not be feasible. If this is the case, we will prioritize the following moderators in our confirmatory moderator analysis:

1. Parents have had one or more children placed in out‐of‐home care before intervention.
2. Parents are at risk because of alcohol/substance abuse and/or mental health issues or parents were themselves placed in out‐of‐home care as children.
3. Parents are at risk because they are young (<20 years).
4. Parents belong to an ethnic minority.
5. Child has a disability.
6. Child age.
7. Child is a boy.

We will add the moderators sequentially in the order described above and stop when the small‐sample adjusted degrees of freedom drops below 4 for any moderator (indicating that we may not be estimating the standard errors well; Tipton, 2015; Tipton & Pustejovsky, 2015). If not all moderators can be included in one meta‐regression, we will also report results from single‐factor subgroup analyses.

The moderator analysis will be conducted separately for ITT and TOT effect sizes. We will report 95% confidence intervals for regression parameters and subgroup analyses. We will estimate the correlations between the covariates and consider the possibility of confounding. Conclusions from meta‐regression analysis will be cautiously drawn and will not solely be based on significance tests. The magnitude of the coefficients and width of the confidence intervals will be taken into account as well.

In general, the strength of inference regarding differences in treatment effects among subgroups is controversial. However, making inferences about different effect sizes among subgroups on the basis of between‐study differences entails a higher risk compared to inferences made on the basis of within‐study differences (see Oxman & Guyatt, 1992). We will therefore use within‐study differences as much as possible. If some studies only report aggregate information (e.g., the proportion of boys in the sample rather than a separate effect estimate for boys and girls), we will use this information to avoid excluding studies from the analysis. In this case, we will also conduct a sensitivity analysis using only within‐study differences. If the two analyses yield different results, we will be cautious in our interpretations and conclusions.

We will also consider the degree of consistency of differences, as making inferences about different effect sizes among subgroups entails a higher risk when the difference is not consistent within the studies (Oxman & Guyatt, 1992).

We will include both studies comparing treatment to control conditions and studies comparing alternative treatments to one another. If there is overlap between treatments examined in treatment‐control and alternative treatment‐designs, analyzing them jointly in a network meta‐analysis may increase statistical power (Chaimani et al., 2022; Salanti, 2012). Comparing the effectiveness of treatments in network meta‐analysis requires assumptions of transitivity and consistency – that the conditions are similar across studies so that condition A is the same, independent of whether it is compared to B or C, B is the same whether it is compared to A or C, and so on (Salanti, 2012). These assumptions seem strong in our setting where it may be difficult to ensure that treatments and, in particular, control conditions are similar enough across studies and settings. However, it may be possible for manual‐based interventions and certain types of control conditions (e.g., no treatment conditions), and if we find such examples, we will consider network meta‐analysis.

If there are enough studies and heterogeneity, we will consider exploratory moderator analyses, which may include other variables (such as intervention components/types, intervention duration and intensity, and researcher conflict of interests) and possibly use machine learning methods to examine heterogeneity (e.g., MetaForest; Van Lissa,  2017). Such analyses should be interpreted cautiously due to the relatively high risk of false discoveries, and the purpose will be to generate new hypotheses about why effect sizes vary that may be tested in future primary studies and meta‐analyses.

#### 3.7.12 Sensitivity analysis

Sensitivity analysis will be carried out by restricting the primary meta‐analysis to a subset of all studies included in the original meta‐analysis and will be used to evaluate whether the pooled effect sizes are robust across components of risk of bias. We will consider sensitivity analysis for each domain of the risk of bias checklists and restrict the analysis to studies with a low risk of bias. Sensitivity analyses with regard to research design and statistical analysis strategies in the primary studies will be an important element of the analysis to ensure that different methods produce consistent results.

As mentioned, if we include studies with clustered assignment of treatment, we will also conduct a sensitivity analysis to examine if these studies have different effect sizes compared to studies with individual assignment of treatment. We test whether the coefficient on a dummy indicating clustered assignment is statistically significant in a meta‐regression otherwise only including an intercept. We will also test if the results are sensitive to the value of *ρ* by running the primary analysis with *ρ* equal to 0 and 0.9 instead of 0.6.

#### 3.7.13 Treatment of qualitative research

We do not plan to include qualitative research.

## CONTRIBUTIONS OF AUTHORS

### Content

Nina Thorup Dalgaard is a psychologist, PhD. Nina has previously worked as both an educational psychologist within a primary school setting and as a clinical child psychologist and thus has knowledge about the socioemotional and cognitive development of children. Currently, Nina is involved in an RCT study exploring the effects of family therapy for foster families in Denmark.

### Systematic review methods

Anja Bondebjerg holds a Master's degree in Sociology and has worked extensively with systematic reviews and research mappings in the fields of education and early childhood education and care. She is knowledgeable regarding the structure and process of conducting systematic reviews.

Anders Bach‐Mortensen is a senior postdoctoral researcher at the Department of Social Policy and Intervention, a Carlsberg Foundation fellow at the Roskilde School of Governance, Roskilde University, and a Research affiliate at The Laboratory for Interdisciplinary Evaluation of Public Policies (LIEPP), Sciences Po, Paris. His research focuses on the outsourcing and marketisation of social care for both adults and children, and he specializes in improving the documentation of this development. He regularly provides methodological expertise on systematic reviews investigating a range of topics.

### Statistical analysis

Jens Dietrichson is an experienced systematic reviewer and methodologist, having completed a number of systematic reviews as well as primary studies in the fields of education and early childhood education and care. He is currently the lead reviewer on three ongoing Campbell Systematic Reviews and is knowledgeable regarding all major facets of meta‐analytic methods and their application.

### Information retrieval

Elizabeth Bengtsen (information specialist) is an experienced research librarian and search specialist who has worked for core research institutions in Denmark for many years, including The Danish National Research Center for the Working Environment. With her profound experience and expertise, Elizabeth will contribute to the review both with her information retrieval skills and her familiarity with systematic review methodology.

## DECLARATIONS OF INTEREST

The authors declare that there are no conflict of interests.

### Preliminary timeframe

We expect to be able to submit the full review aproximately 1 year after publication of the protocol.

### Plans for updating this review

The review will be updated if funding is available after a period of 5 years. The review team is responsible for future updates.

## SOURCES OF SUPPORT

### Internal sources

- VIVE—The Danish Center for Social Science Research, Denmark

### External sources

- No sources of support provided

## Supporting information

Supporting information.
